# Assessment of Bacterial Communities Associated With the Skin of Costa Rican Amphibians at La Selva Biological Station

**DOI:** 10.3389/fmicb.2018.02001

**Published:** 2018-09-03

**Authors:** Juan G. Abarca, Gabriel Vargas, Ibrahim Zuniga, Steven M. Whitfield, Douglas C. Woodhams, Jacob Kerby, Valerie J. McKenzie, Catalina Murillo-Cruz, Adrián A. Pinto-Tomás

**Affiliations:** ^1^Centro de Investigación en Estructuras Microscópicas, Universidad de Costa Rica, San Pedro, Costa Rica; ^2^Department of the Geophysical Sciences, University of Chicago, Chicago, IL, United States; ^3^Departamento de Bioquímica, Escuela de Medicina, Universidad de Costa Rica, San Pedro, Costa Rica; ^4^Centro de Investigación en Biología Celular y Molecular, Universidad de Costa Rica, San Pedro, Costa Rica; ^5^Department of Conservation and Research, Zoo Miami, Miami, FL, United States; ^6^Department of Biology, University of Massachusetts, Boston, MA, United States; ^7^Smithsonian Tropical Research Institute, Panama City, Panama; ^8^Department of Biology, University of South Dakota, Vermillion, SD, United States; ^9^Department of Ecology and Evolutionary Biology, University of Colorado, Boulder, Boulder, CO, United States

**Keywords:** amphibian, microbiota, La Selva Biological Station, bacterial communities, *Batrachochytrium dendrobatidis*

## Abstract

Amphibian skin is a suitable environment for rich communities of microorganisms, both beneficial and detrimental to the host. The amphibian cutaneous microbiota has been hypothesized to play an important role as symbionts, protecting their hosts against disease. Costa Rica has one of the most diverse assemblages of amphibians in the world and we know very little about the microbiota of these tropical animals. For comparison with other studies, we explore the diversity of the skin bacterial communities employing16S rRNA amplicon sequencing of swab samples from twelve species of frogs at La Selva Biological Station in Sarapiquí, Heredia province. The predominant phylum detected in our studies was Proteobacteria, followed by Bacteroidetes and Actinobacteria, with these three phyla representing 89.9% of the total bacterial taxa. At the family level, Sphingobacteriaceae and Comamonadaceae were highly represented among samples. Our results suggest that host species and host family are significant predictors of the variation in microbiota composition. This study helps set the foundation for future research about microbiota composition and resilience to unfavorable conditions, leading to improvement in managing strategies for endangered amphibian species.

## Introduction

Dramatic declines in amphibian populations around the world have been a subject of research in recent years. Since the 1980s, amphibian populations have declined to the point where 42% of the 7854 species described worldwide (AmphibiaWeb, 2018) have experienced some form of population declines (Stuart et al., 2004; Whittaker et al., 2013). Population declines in amphibians have been attributed to several factors, mainly related to human activities such as habitat destruction, pesticide abuse and climate change, among others (Whitfield et al., 2007; Kerby et al., 2011). However, population declines in protected areas, so called “enigmatic declines,” have drawn the attention to the emerging disease chytridiomycosis, caused by the fungus *Batrachochytrium dendrobatidis* (*Bd*) (Lips et al., 2006; Whitfield et al., 2007, 2012; Lips, 2016).

Costa Rica has become a focus for amphibian research. In the late 1990s, a study revealed declines of up to 40% of amphibian populations in the Monteverde Cloud Forest Reserve (Pounds et al., 1997). Also, Lips (1998) documented population declines in Costa Rican amphibians at Las Tablas. Another hotspot for amphibian studies in Costa Rica is La Selva Biological Station in Heredia Province. This is a 16 km^2^ private protected area, managed by the Organization of Tropical Studies, where amphibians have been studied for almost 40 years. Whitfield and collaborators analyzed data collected for over 35 years and they found that all species of terrestrial amphibians declined approximately 75% in this site. Interestingly, that study showed a correlation between population declines and a climate driven reduction of the quantity of leaf litter, but no correlation to *Bd* emergence was detected (Whitfield et al., 2007).

Over the last years, next generation sequencing technologies allowed scientists to explore oceans, soils, humans and a variety of host related microbiota (Jani and Briggs, 2014). These technologies can be implemented to study amphibians, specifically their skin microbiota. Skin is especially important for amphibian physiology; it is responsible for the exchange of respiratory gasses and osmoregulation (Voyles et al., 2009). Furthermore, the skin is in continuous exposure to microorganisms in the environment, hence these potentially symbiotic microbes may be involved in different physiological processes and interacting with pathogens such as *Bd* (Madison et al., 2017). Knowledge of the ecology of bacterial communities associated with amphibians has only a few years of development (Bletz et al., 2017a). Previous research showed that bacterial communities of amphibian skin were significantly different to their environments and one of the most important factors driving the structure of the microbiota was host species (McKenzie et al., 2012; Kueneman et al., 2013; Sabino-Pinto et al., 2016). More recently, it has been suggested that environmental factors may also play an important role in the variation of the amphibian bacterial microbiota (Muletz et al., 2017; Bird et al., 2018; Varela et al., 2018). It has also been reported that bacterial communities can vary according to geography (Abarca et al., 2018; Rebollar et al., 2018), ecomorphs (Bletz et al., 2017a) and ontogeny (Longo et al., 2015).

The discovery of bacteria with antagonistic activity to *Bd* (Harris et al., 2006, 2009) opens the possibility of employing new strategies to control the pathogen; however, due to the rapid loss of species and habitat, it is necessary to speed up the search for beneficial bacteria to be employed in captivity or in wildlife (Flechas et al., 2017). To accelerate this search, the use of culture independent techniques can be a very efficient tool to describe local bacterial communities (Rebollar et al., 2016a). Due to the variation in the inhibitory response of bacteria to *Bd* (Antwis and Harrison, 2018), and because skin bacterial communities differed strongly from those of the surrounding environment (Bletz et al., 2017a), it has been recommended to implement mitigation strategies at the local level, using bacteria to counteract the circulating pathogen in the same region (Flechas et al., 2017). The aim of this study is to explore and compare the diversity of the skin bacterial communities of twelve tropical amphibian species at La Selva Biological Station in Costa Rica. Further, these communities are compared to previous studies with other amphibian species and were examined for potential anti-*Bd* function. Our goal is to describe this host-associated microbiota and set the foundation to understand microbiota composition and potential interaction with *Bd*, as well as to describe host specific relationships between amphibians and microbes in Costa Rica.

## Materials and Methods

### Study Site, Species and Sampling

All sampling was performed at La Selva Biological Station in Sarapiquí, Heredia Province, northeastern Costa Rica (10°26′ N, 83°59′ W), between January 18th–19th, 2013. La Selva is a private biological reserve with elevation ranging from 35 m to 137 m.a.s.l., managed by the Organization for Tropical Studies for over 45 years and has been described as lowland wet forest. About 73% of the station territory is primary forest, the rest of the reserve includes forests regenerating from pasture and other agricultural uses (Whitfield et al., 2012). We sampled 66 specimens within twelve species of frogs from nine different families. These included 8 terrestrial species, 3 arboreal and one arboreal-aquatic species (**Table 1** and **Supplementary Table 1**). We sampled by visual encounter on the trails, every specimen was kept in individual clean plastic bags. Once in the lab, each amphibian individual was handled with new sterile nitrile gloves. Each frog was washed for 7 s using 50 ml of sterile distilled water. Sterile swabs were rubbed 10 times on the ventral and dorsal areas and 5 more times on each side (Boyle et al., 2004; Bird et al., 2018). After skin swabbing, frogs were released to their natural environment in less than 3 h. The swabs were placed directly in dry labeled 1.5 ml tubes, transported to the laboratory the same day in an ice-filled container and stored at -80°C until DNA was extracted 2 days later.

**Table 1:** Amphibian samples processed for skin bacteria community analysis at La Selva Biological Station.

Family	Species	Common name	Individuals	Habitat
Craugastoridae	*Craugastor bransfordii*	Bransford’s Litter Frog	5	Terrestrial
	*Craugastor fitzingeri*	Common Rain Frog	10	Terrestrial
	*Craugastor megacephalus*	Veragua Robber Frog	2	Terrestrial
Bufonidae	*Rhinella horribilis*	Cane toad	1	Terrestrial
	*Rhaebo haematiticus*	Truando Toad	10	Terrestrial
Phyllomedusidae	*Agalychnis callidryas*	Red Eyed Tree Frog	4	Arboreal
Hylidae	*Smilisca manisorum*	Mexican Tree Frog	1	Aquatic-arboreal
Strabomantidae	*Pristimantis ridens*	Pygmy Robber Frog	2	Arboreal
Dendrobatidae	*Oophaga pumilio*	Strawberry Poison Frog	25	Terrestrial
Eleutherodactylidae	*Diasporus diastema*	Dink Frog	2	Arboreal
Leptodactylidae	*Leptodactylus savagei*	Jungle Smoking Frog	1	Terrestrial
Microhylidae	*Hypopachus pictiventris*	Southern Narrow-mouthed Toad	2	Terrestrial

### DNA Extraction and Sequencing

DNA extractions were performed using the MOBIO PowerSoil Extraction kit (MO BIO, Carlsbad, CA, United States) according to manufacturer’s protocols, with the following modifications: samples were incubated at 65°C after the addition of reagent C1, the powerbead tubes were vortexed horizontally for 2 min (Lauber et al., 2008), and the reagent C6 was left on the filter for 5 min before the final elution. Eighteen negative controls were extracted from sterile swabs. DNA extractions were transported in ice packs to the University of Colorado Boulder and stored at -80°C for 15 days until barcoding addition. DNA was sequenced 3 weeks later. PCR reactions contained 11 μL PCR water, 10 μL of 5 Prime Master Mix (5 PRIME GmbH), 1.0 μL each of the forward and reverse primers (at 10 μM), 1.0 μL MgCl_2_, and 1.0 μL genomic DNA. PCR primers (F515/R806) were used to target the V4 region of 16S rRNA. This primer pair amplifies the 533–786 region in *Escherichia coli* strain 83972 genome sequence (Greengenes accession no. prokMSA_id:470367). The reverse PCR primer contained a 12-base Golay error-correcting barcode originally developed by Caporaso et al. (2011). PCR conditions consisted of an initial denaturation step of 94°C for 3 min, followed by 35 cycles at 94°C for 45 s, 50°C for 60 s, and 72°C for 90 s; and final extension of 10 min at 72°C. From 18 negative controls, five yielded PCR bands, these samples were sequenced as described below and filtered out from the sequence analysis. Each sample was amplified in triplicate and combined. Amplicons were quantified using the Quant-IT Picogreen dsDNA reagent in 1X TE buffer. A composite sample for sequencing was created by combining equimolar ratios of amplicons from the individual samples and cleaned using the MO BIO UltraClean PCR clean up DNA purification kit. Purity and DNA concentration were determined using a NanoDrop spectrophotometer (Thermo Scientific). Samples were processed on an Illumina MiSeq Sequencer (Illumina Inc., San Diego, CA, United States) at the Next Generation Sequencing Facility located at the University of Colorado Boulder. The resulting raw sequences for each sample were deposited as Sequence Read Archive (SRA) in the NCBI BioProject ID PRJNA470570.

### Sequence Analyses

Quantitative Insights Into Microbial Ecology (QIIME v1.9.0) (Caporaso et al., 2010b) was employed for all sequence analyses. Sequences were filtered for quality and assigned to their respective sample using default settings. The resulting 3,551,436 reads were clustered into 89,504 operational taxonomic units (OTUs) according to the subsampling open reference protocol using the Greengenes May 2013 97% reference sequences^1^. The reads that did not match the reference dataset were clustered into *de novo* OTUs. Chimeric reads, reads matching archaea and mitochondria, as well as low abundance OTUs (lower than 0.00005 of the total read count) were removed from downstream analyses. We detected 116 different OTUs in the negative controls, of which 10 had abundance percentages greater than 1.0%, these OTUs were filtered and removed from further analyses. Samples were subsequently rarefied at 7,800 reads per sample to normalize read counts across samples. Sequences were aligned to the Greengenes (v. gg_13_8) reference alignment using PyNast (Caporaso et al., 2010a). We then constructed a phylogenetic tree using FastTree according to standard procedures within QIIME. Taxonomic summaries at the phylum and genus levels were built by using QIIME’s plot tables L2 and L6, respectively, using the melt function in the RESHAPE2 R package (Wickham, 2007). Then we calculated the core OTUs (OTUs present in 90% of the samples) and the more abundant OTUs, a heatmap of these OTUs was built using heatmap.3 function in R. We determined Shannon diversity index and Faith’s phylogenetic diversity (PD_whole_tree) and employed a parametric ANOVA test to compare the means of the estimators by species and habitat.

Beta diversity analysis was performed with a multidimensional scaling plot (PCoA) using the weighted Unifrac distances and plotted with the ordinate function in the PHYLOSEQ R packages (McMurdie and Holmes, 2013). We also performed analysis of similarity (ANOSIM) and permutational multivariate analysis of variance (PERMANOVA) to examine the contribution of host species, host family and habitat in the variation of bacterial community composition. Additionally, we employed an ADONIS test to examine the simultaneous effects of host species and habitat. We compared the diversity of tropical versus temperate amphibian skin microbiota using our Costa Rica data set and similar datasets of amphibians from California and Virginia (Kueneman et al., 2013; Loudon et al., 2014). Finally, the OTU table was filtered to retain only reads matching sequences of bacteria from the Antifungal Isolates Database (Woodhams et al., 2015) that were previously shown to inhibit the growth of *Bd*. We calculated the proportion of these putatively anti-*Bd* bacteria for each sample and tested for differences among species and between habitat types. Non-parametric tests were employed in IBM SPSS Statistics v21 because data did not meet the assumptions of equality of variance.

## Results

**FIGURE 1:**
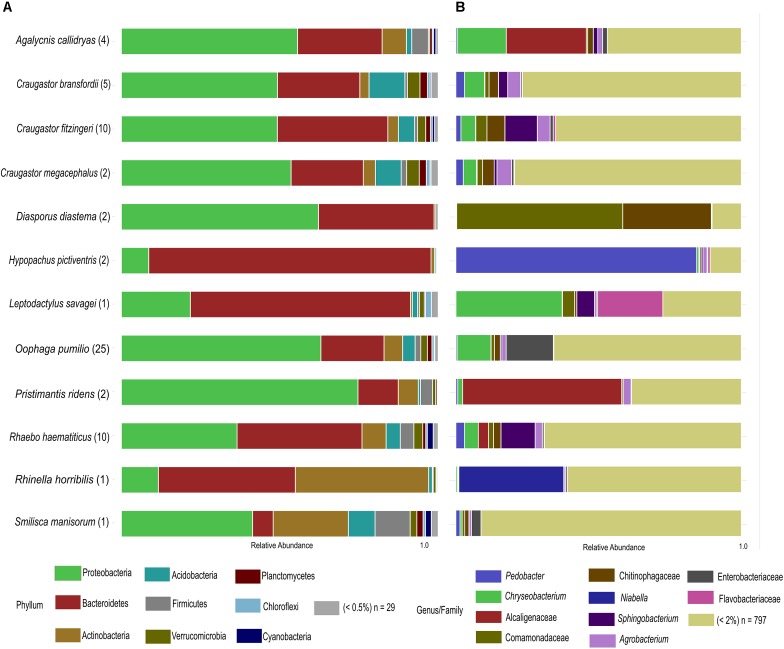
Taxonomic profile of skin bacterial communities of Costa Rican frogs from La Selva Biological Station. **(A)** Phylum-level relative abundance profiles of the host-associated skin bacterial communities. OTUs with abundances lower than 0.5% within each Phylum were clustered together. **(B)** Genus/family level relative abundance profiles of the frog-associated skin bacterial communities. OTUs with percentages lower than 2% of the microbiota composition within species were clustered together. The number of individuals sampled for each species is shown in parenthesis.

After rarefaction, we obtained a total of 2,621,143 good quality sequences and 6330 OTUs distributed in 38 bacterial phyla (**Figure 1A**). Eight out of the twelve species under study were dominated by the phylum Proteobacteria (43.95% of the total OTUs). Other abundant phyla were Bacteroidetes (35.59%) and Actinobacteria (8.79%), while Acidobacteria (3.91%), Firmicutes (2.61%), Verrucomicrobia (1.79%) and Planctomycetes (1.04%) were other phyla represented in at least 1% of the OTUs. Among the four species that were not dominated by Proteobacteria, *Hypopachus pictiventris*, *Leptodactylus savagei*, and *Rhaebo haematiticus* presented a majority of Bacteroidetes OTUs, with 89.02, 69.47, and 39.40%, respectively. The bacterial community associated with *Rhinella horribilis* showed a dominance of Bacteroidetes (43.23%) and Actinobacteria (42.08%), besides this species, only *Smilisca manisorum* presented a relative abundance of actinomycete OTUs above 10% (23.72%).

We identified 463 bacterial families in our dataset. In terms of relative abundance of OTUs, the Sphingobacteriaceae family presented the highest percentage (13.82%), followed by Comamonadaceae (9.08%), Weeksellaceae (8.14%), Chitinophagaceae (7.95%), and Alcaligenaceae (7.46%) (**Supplementary Table 2**). The phylogenetic distribution at family and genus level was noteworthy (**Figure 1B**), for example, showing the dominance of *Pedobacter* in the skin microbiota of *H. pictiventris* (84.34%). In *C. bransfordii* the bacterial community was weakly dominated by OTUs of the genus *Chryseobacterium* (7.00%) and *Agrobacterium* (4.51%). The bacterial community of *C. fitzingeri* was dominated by OTUs of the genus *Sphingobacterium* (11.34%) and one OTUs in Chitinophagaceae family (6.28%); *C. megacephalus* had a bacterial community dominated weakly by *Agrobacterium* (5.10%). The bacterial community of *O. pumilio* had a dominance of OTUs related to Enterobacteriaceae (16.54%) and the bacterial community of *R. haematiticus* was dominated by the genus *Sphingobacterium* (11.98%). The bacterial community of *A. callidryas* was dominated by Alcaligenaceae OTUs (28.04%). The Cane toad, *R. horribilis*, showed an abundance of OTUs in the genus *Niabella*, family Chitinophagaceae (36.75%). *P. ridens* was dominated by OTUs in the family Alcaligenaceae (55.7%), while the *L. savagei* community was dominated by *Chryseobacterium* (37.21%) followed by another OTU belonging to the family Flavobacteriaceae (22.82%).

**FIGURE 2:**
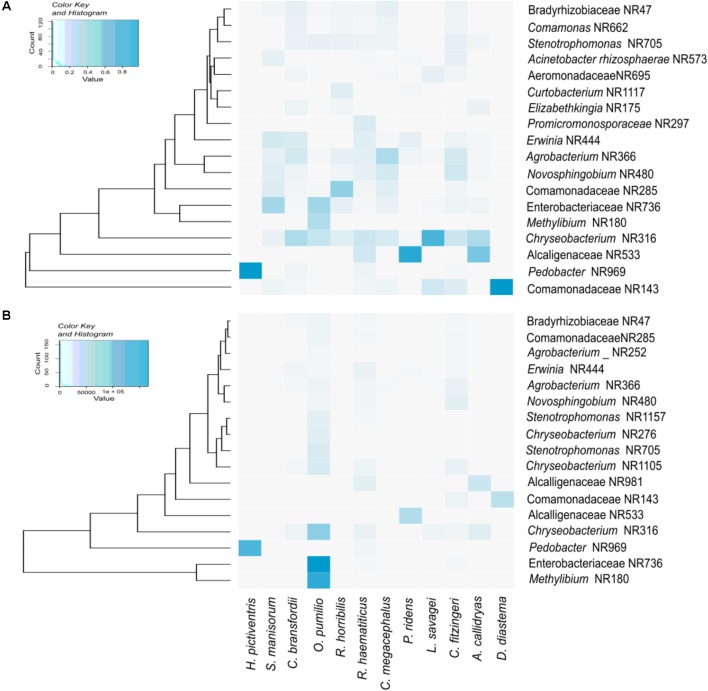
Heatmaps depicting the core skin bacteria present in 90% of samples **(A),** and the OTUs with higher relative abundance **(B)**.

**Figure 2** shows a heatmap of the core microbiota (OTUs that are present in 90% of the samples), as well as the OTUs that have relative abundances significantly different between the bacterial communities. All amphibian species shared OTUs belonging to the genera *Chryseobacterium* and *Pedobacter*, as well as several OTUs assigned to the families Alcaligenaceae, Comamonadaceae and Rhizobiaceae. The three most abundant OTUs detected in this study were assigned to the family Enterobacteriacea and to the genera *Methylibium* and *Pedobacter*.

The relative abundance of OTUs varied among individuals within each species, as follows: In *O. pumilio* (*n* = 25), 12 individuals were dominated by Enterobacteriaceae OTUs, 7 individuals were dominated by *Methylibium* OTUs, while the other 6 had higher abundance of Chitinophagaceae and *Stenotrophomonas*. In *C. fitzingeri* (n = 10), 3 individuals were dominated by *Sphingobacterium* OTUs, 2 by *Chryseobacterium* OTUs and the 5 remaining individuals each had a different most abundant OTU. Regarding *R. haematiticus* (*n* = 10), 4 individuals were dominated by *Sphingobacterium* OTUs, 2 by *Chryseobacterium* OTUs and the 4 remaining individuals each had a different most abundant OTU. In two species, *C. bransfordii* (n = 5) and *A. callidryas* (n = 4), all individuals were dominated by a different OTU. Finally, all species with 2 individuals sampled had the same dominant OTU (*G. pictiventris, P. ridens* and *D. diastema*), except for *C. megacephalus*, in which one individual was dominated by *Chryseobacterium* and the other by *Agrobacterium* OTUs.

**FIGURE 3:**
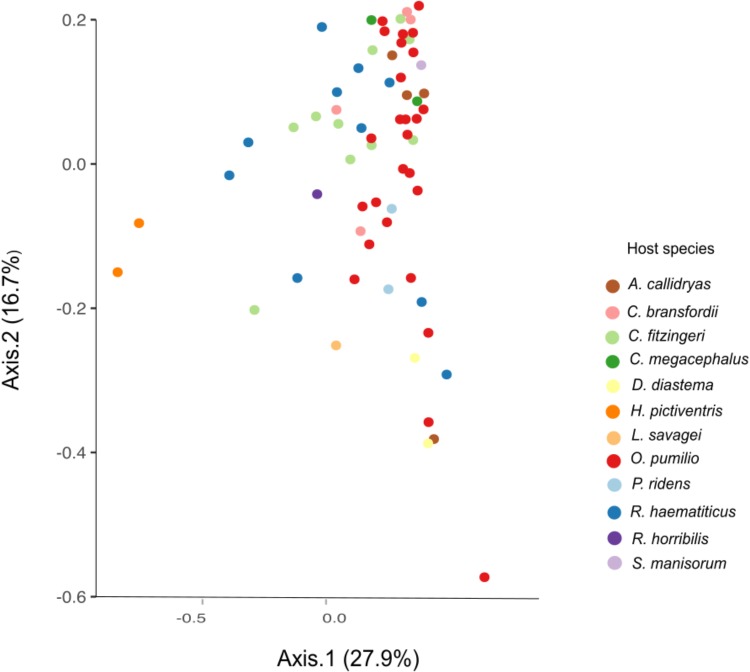
Beta diversity PCoA by weighted UNIFRAC of amphibian skin bacterial communities from La Selva Biological Station. Axes show the major components that explain 44.6% of the differences between samples.

**FIGURE 4:**
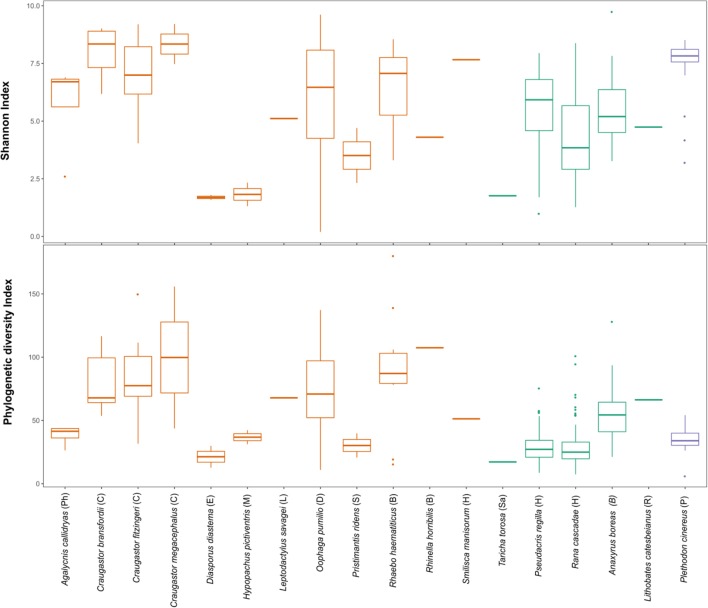
Shannon index and Faith Phylogenetic diversity of amphibian skin bacterial communities from Costa Rica (orange), California (green) and Virginia (purple). Letters in parenthesis represent family: **Ph**, Phyllomedusidae; **C**, Craugastoridae; **E**, Eleutherodactylidae; **M**, Microhylidae; **L**, Leptodactylidae; **D**, Dendrobatidae; **S**, Strabomantidae; **B**, Bufonidae; **H**, Hylidae; **Sa**, Salamandridae; **R**, Ranidae; **P**, Plethodontidae.

The beta diversity analysis did not show clear clustering among the different bacterial communities; however, some trends are worth noticing. **Figure 3** shows *H. pictiventris* communities separately from the rest of the frogs. Other species did cluster together such as *D. diastema*, while some samples of *O. pumilio* and one of *A. callidryas* grouped close to them. Most *O. pumilio* samples are widely distributed around the plot, indicating great variation within this species. Beta-diversity analysis showed that host species (ANOSIM *R* = 0.416, *p* = 0.01; PERMANOVA PseudoF = 3.388, *p* = 0.001) and host family (ANOSIM *R* = 0.392, *p* = 0.01; PERMANOVA PseudoF = 3.751, *p* = 0.001) are significant predictors of the variation in bacterial community composition. Contrary, host habitat did not show significant results (ANOSIM *R* = 0.171, *p* = 0.11; PERMANOVA PseudoF = 1.866, *p* = 0.0599). The combined effect of host species and host habitat also influenced significantly the variation in bacterial community composition (ADONIS *F* = 3.3884, *p* = 0.001). Regarding alpha-diversity, both Shannon and phylogenetic diversity indexes presented similar results (**Figure 4**): they did not vary significantly between amphibian species (*P* > 0.005 in both indexes). Frogs of the genus *Craugastor*, *O. pumilio*, *R. haematiticus* and *S. baudinii* have the most diverse skin bacterial communities. On the other hand, *D. diastema, P. ridens* and *H. pictiventris* showed lower diversity (**Supplementary Table 3**). Regarding habitat, there was a significant variation between indexes according to habitat (*p* < 0.005 in both), showing a higher diversity associated to terrestrial species when compared to arboreal species. On the other hand, there were no differences between diversity indexes when we compared terrestrial species versus aquatic species or arboreal species versus aquatic ones (*p* > 0.005 in all indexes).

**FIGURE 5:**
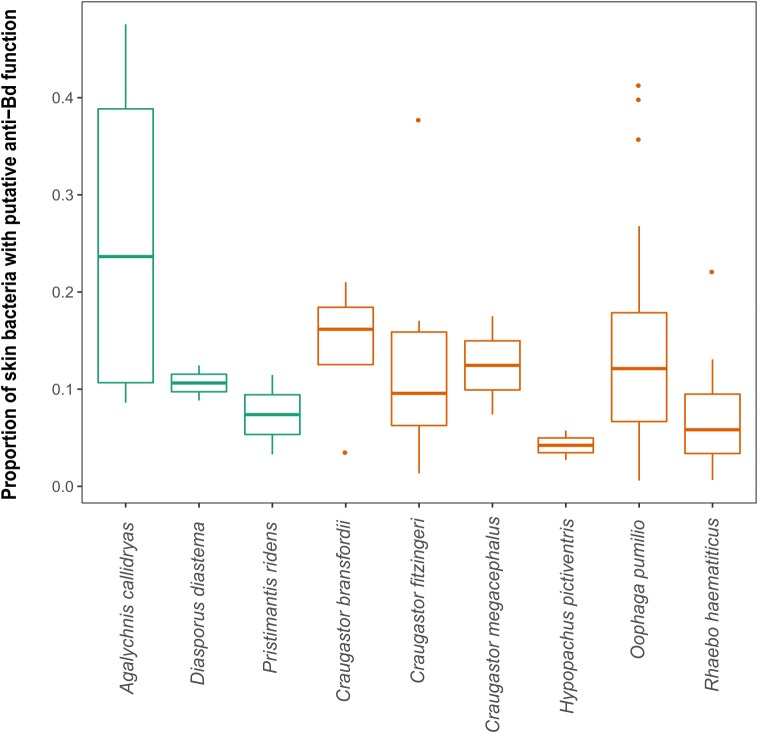
Proportion of putatively anti-*Bd* bacteria from the Antifungal Isolates Database. Note that 9 of the 12 species are shown after removing species with only one sample. No significant differences were found in proportion of putatively anti-*Bd* bacteria among species or between arboreal (orange) and terrestrial (green) habitat types.

Finally, we compared tropical and temperate species, but no significant latitudinal differences were found in alpha diversity; furthermore, two overlapping families, Bufonidae and Hylidae, also presented similar alpha diversity values (**Figure 5**). The proportion of putatively anti-*Bd* bacteria showed no significant differences among species (Kruskal–Wallis test, *p* = 0.149) or between arboreal and terrestrial habitat types (Mann–Whitney *U*-test, 0.714). However, all 12 species and all samples contained putatively anti-*Bd* bacterial taxa with an average of 13.3% of the 16S rRNA sequencing reads (**Figure 5**).

## Discussion

Amphibian skin is an important organ for electrolyte and gas exchange with the environment, and many diseases and contaminants can affect amphibians through it (Campbell et al., 2012). Unfortunately, very little is known about amphibian-associated microbiota in a biodiversity hotspot that has suffered from severe amphibian decline such as Costa Rica. To start bridging this knowledge gap, we studied the skin microbiota of several frog species at La Selva Biological Station. We found that skin bacterial communities differ among amphibian species. Species-level differences in the amphibian skin microbiota have been previously documented in temperate (McKenzie et al., 2012; Kueneman et al., 2013; Walke et al., 2014) and tropical systems (Flechas et al., 2012; Belden et al., 2015; Rebollar et al., 2016b). Furthermore, we found that host family is also a good predictor of bacterial community variation. In tropical regions there are more frog families occupying different microhabitats, this is not the case for temperate zones, where host family was not a strong driver of microbiota when compared to host habitat (Kueneman et al., 2013).

It has been reported that host ecology and habitat are more important factors of variation than host taxonomy (Bletz et al., 2017a; Muletz et al., 2017; Bird et al., 2018), and that most amphibian microbiota comes from the environment (Rebollar et al., 2016b). Despite this, we did not find that habitat was a predictor of variation in the bacterial community of the population under study. This result may be caused by the limited sampling range, or it could reflect amphibian ecology at La Selva, where species transition between different habitats. For example, *O. pumilio* is principally terrestrial but they climb trees to bring tadpoles to bromeliads, *R. haematiticus* can be seen in the forest floor as well as in rivers or even in low vegetation and *C. fitzingeri* can be observed in forest leaf litter, vegetation, near rivers and open areas with pastures (Savage, 2002). However, further research is needed to confirm these results, including analyzing more species from aquatic habitats.

Proteobacteria was the most commonly detected phylum and was more abundant in leaf litter species. This is the main phylum recovered from soils (Kim et al., 2014), tree leaves (Kembel et al., 2014) and amphibians (Belden et al., 2015; Bletz et al., 2017a; Varela et al., 2018) in the tropics. The other three dominant phyla agree with the main four phyla of bacteria associated with amphibians in temperate or tropical areas (Lauer et al., 2008; McKenzie et al., 2012; Walke et al., 2014; Becker et al., 2014; Belden et al., 2015). However, we found that Comamonadaceae and Alcaligenaceae were the most common Proteobacteria families in the bacterial communities of the amphibians sampled, in contrast with other studies in both tropical and temperate zones that identified Pseudomonadaceae as the most abundant family (Lam et al., 2010; Rebollar et al., 2016b, 2018).

Bacteroidetes was the second most abundant phylum in skin of the frogs at La Selva. This phylum is also widely distributed in the environment, including soils, sediments and sea water (Thomas et al., 2011). Bacteroidetes is common in four aquatic and terrestrial frog species. Important genera within this phylum present in the microbiota of amphibians at La Selva were *Pedobacter*, *Chryseobacterium* and *Niabella*. High dominance of *Pedobacter* OTUs was found in *H. pictiventris.* The abundance of this genus is important because it has been shown to have inhibitory activity against *Bd* (Harris et al., 2006; Lauer et al., 2008). *Pedobacter* has been isolated from multiple habitats and it can dominate aerobic communities in different aquatic and terrestrial environments, due to its high metabolic diversity (Gordon et al., 2009). *H. pictiventris* spends most of its life underground but in the breeding season they congregate in puddles in the forest (Savage, 2002). This sudden change of microclimate could affect the skin bacterial community allowing *Pedobacter* to thrive over other genera. However, further research is needed to establish whether these two organisms maintain a symbiotic relationship.

*Craugastor* spp., *R. haematiticus, R. horribilis*, and *O. pumilio* had higher overall bacterial diversity compared with the rest of species; *P. ridens* and *H. pictiventris* have the least bacterial diversity. At La Selva, alpha diversity metrics as Shannon and Phylogenetic diversity indexes show that typical leaf-litter dwelling families (Craugastoridae, Bufonidae and Dendrobatidae) have greater bacterial diversity than arboreal or aquatic families such as Hylidae, Phyllomedusidae, Leptodactylidae, Microhylidae or Eleutherodactylidae. It is possible that more soil bacteria can colonize amphibians in the leaf litter than in arboreal or aquatic habitats, this pattern agrees with previous studies (Belden et al., 2015; Rebollar et al., 2016b; Bletz et al., 2017a).

Our results show differences with similar studies performed in nearby Panama. In those studies, different frog species were analyzed (except for *C. fitzingeri* and *A. callidryas*), suggesting that differences in bacterial composition between countries may be due to different host species tested. One important difference is that Actinobacteria was the second most abundant phylum in frogs of Panama (Belden et al., 2015; Rebollar et al., 2016b), while in Costa Rica this group was in third place. For shared species, however, the alpha diversity indexes appear to be similar, with higher values for *C. fitzingeri*. The microbiota of *C. fitzingeri* from La Selva is enriched with OTUs of the Sphingobacteriaceae family, while those of Panama are enriched with other families such as Comamonadaceae and Moraxellaceae (Rebollar et al., 2018). The Shannon index of *C. fitzingeri* in Costa Rica is similar to that of *C. fitzingeri* in *“Bd-naive”* sites in Panamá (Rebollar et al., 2016b), although La Selva is a “*Bd*-endemic” site where it has been reported that *C. fitzingeri* along with other species of amphibians carry *Bd* (Whitfield et al., 2012). Additionally, in our study, in every species with more than 4 amphibians sampled, the most abundant OTU was not the same in all of them. Both observations suggest that there are intraspecific variations in amphibian bacterial communities, these variations are possibly associated with environmental factors, presence of *Bd* or internal population variations of amphibian species, as has been reported by other studies (Muletz et al., 2017; Abarca et al., 2018; Rebollar et al., 2018).

We found similarities in Shannon diversity index and phylogenetic diversity between temperate zones and tropical zones. At bacterial composition level, temperate zones frogs appear to have more Firmicutes than tropical ones (Kueneman et al., 2013; Loudon et al., 2014; Belden et al., 2015). It is important to point out that the temperate studies included mostly aquatic species, and only one terrestrial family: Bufonidae, while at La Selva we studied mostly terrestrial and arboreal species; therefore, we can only compare two families between Costa Rica and temperate zones: Bufonidae and Hylidae. Those families showed similar richness and phylogenetic diversity. In Bufonidae, Kueneman et al. (2013) found similar abundance of three phyla, the same found in *R. horribilis* at La Selva. Both in Kueneman et al. (2013) and in Belden et al. (2015) the aquatic temperate species was *Lithobates catesbeianus*, and they found different microbiota composition between tropical and temperate populations. To establish a better comparison, it is necessary to characterize the bacterial community of a fully aquatic tropical frog, ideally in the Family Ranidae. Even though the tropics present very different conditions than temperate zones, it is possible that the skin texture and habits of certain species may represent a more homogeneous microhabitat for some bacterial genera regardless of geographical location.

We recovered several sequences related to putatively anti-*Bd* bacterial strains in the Antifungal Isolates Database (Woodhams et al., 2015). This database has been used to determine proportions of OTUs of anti-*Bd* bacteria among other species of amphibians (Kueneman et al., 2016), as well as to study associations between the degrees of infection and the presence of OTUs related to inhibitory bacteria (Jani et al., 2017). *Chryseobacterium* and *Pseudomonas* comprised the majority of putatively anti-*Bd* bacteria from all species examined. These two genera have been reported as the most abundant among inhibitory *Bd* bacteria within their phylum (Park et al., 2014; Becker et al., 2015). In this study over 13% of sequences were related to putatively anti-*Bd* strains. This percentage of putatively anti-*Bd* bacteria is similar to that found using culture-based techniques in Costa Rican amphibians (11.1% in cell-free supernatant assays) (Madison et al., 2017). The presence of OTUs associated with putatively anti-*Bd* bacteria suggests the existence of bacteria that can help fight the disease, but this is still a hypothetical value. For this reason, inhibition tests with cultivable bacteria from each amphibian species (Antwis et al., 2015; Bletz et al., 2017b; Burkart et al., 2017) are necessary to confirm if some amphibians indeed carry higher proportions of anti *Bd* bacteria.

Understanding the microbial community in each region is one of the first steps to implement mechanisms to mitigate chytridiomycosis, including employing probiotic bacteria (Woodhams et al., 2016). Here we find that skin bacterial community of amphibians at La Selva varies according to species and family. Within the bacterial community there are potential symbiotic bacteria that should be the focus of future studies. It is important to know and describe local bacterial communities associated with amphibian skin to understand the mechanisms of interaction between these microorganisms and thus implement efficient bioaugmentation strategies (Flechas et al., 2012). This work represents an effort to characterize the bacterial communities of amphibians in Costa Rica. La Selva Biological Station is a tropical forest research center of worldwide recognition, and this research can serve as a foundation for further studies aimed at designing mitigation strategies to control the threat of *Bd* and human-associated factors as drivers of amphibian extinction.

## Ethics Statement

This study was carried out in accordance with the recommendations of the Institutional Commission on Biodiversity at the University of Costa Rica. Our work with amphibians has been reviewed and approved by the Animal Care and Ethics Committee from the University of Costa Rica (Resolution CICUA-029-15).

## Author Contributions

SW, JK, VM and AP-T conceived and designed the experiments. JA, GV, IZ, and CM-C performed the experiments. JA, GV, IZ, SW, DW, JK, and VM analyzed the data. JK, VM, and CM-C contributed reagents, materials, and analysis tools. JA, GV, IZ, DW, VM, and AP-T wrote the paper. SW, DW, JK, and CM-C reviewed draft on the paper.

## Conflict of Interest Statement

The authors declare that the research was conducted in the absence of any commercial or financial relationships that could be construed as a potential conflict of interest. The reviewer MV declared a past co-authorship with several of the authors VM and DW to the handling Editor.

## Footnotes

1 greengenes.secondgenome.com
